# Reduced IFNL1 and/or IFNL2, but not IFNL3 is associated with worse outcome in patients with COVID-19

**DOI:** 10.1093/cei/uxae047

**Published:** 2024-07-02

**Authors:** Elena Woods, Adriana Mena, Sophie Sierpinska, Emily Carr, STTAR Bioresource, Richard Hagan, John Crowley, Colm Bergin, David Clark, Caroline Brophy, Derek Macallan, Clair M Gardiner

**Affiliations:** School of Biochemistry and Immunology, Trinity Biomedical Research Institute, Trinity College Dublin, Dublin 2, Ireland; School of Biochemistry and Immunology, Trinity Biomedical Research Institute, Trinity College Dublin, Dublin 2, Ireland; Institute for Infection and Immunity, St George’s, University of London, Cranmer Terrace, London, UK; Institute for Infection and Immunity, St George’s, University of London, Cranmer Terrace, London, UK; STTAR Bioresource, CRF, St. James’s Hospital, Dublin, Ireland; National Histocompatibility and Immunogenetics Reference Laboratory, National Blood Centre, Dublin, Ireland; National Histocompatibility and Immunogenetics Reference Laboratory, National Blood Centre, Dublin, Ireland; Department of Infectious Diseases, St. James’s Hospital, Dublin, Ireland; Institute for Infection and Immunity, St George’s, University of London, Cranmer Terrace, London, UK; School of Computer Science and Statistics, Trinity College Dublin, Dublin 2, Ireland; Institute for Infection and Immunity, St George’s, University of London, Cranmer Terrace, London, UK; Infection and Immunity Clinical Academic Group, St George’s University Hospitals NHS Foundation Trust, London, UK; School of Biochemistry and Immunology, Trinity Biomedical Research Institute, Trinity College Dublin, Dublin 2, Ireland

**Keywords:** interferon lambda, IFNL1, IFNL2, IFNL3, IL28A, IL28V, COVID-19, human

## Abstract

The recent pandemic was caused by the emergence of a new human pathogen, SARS-CoV-2. While the rapid development of many vaccines provided an end to the immediate crisis, there remains an urgent need to understand more about this new virus and what constitutes a beneficial immune response in terms of successful resolution of infection. Indeed, this is key for development of vaccines that provide long lasting protective immunity. The interferon lambda (IFNL) family of cytokines are produced early in response to infection and are generally considered anti-viral and beneficial. However, data regarding production of IFNL cytokines in coronavirus disease 2019 (COVID-19) patients is highly variable, and generally from underpowered studies. In this study, we measured all three IFNL1, IFNL2, and IFNL3 cytokines in plasma from a well characterized, large COVID-19 cohort (*n* = 399) that included good representation from patients with a more indolent disease progression, and hence a beneficial immune response. While all three cytokines were produced, they differed in both the frequency of expression in patients, and the levels produced. IFNL3 was produced in almost all patients but neither protein level nor IFNL3/IFNL4 single nucleotide polymorphisms were associated with clinical outcome. In contrast, both IFNL1 and IFNL2 levels were significantly lower, or absent, in plasma of patients that had a more severe disease outcome. These data are consistent with the concept that early IFNL1 and IFNL2 cytokine production is protective against SARS-CoV-2 infection.

## Teaser

Circulating interferon lambda cytokines (IFNL1, IFNL2, and IFNL3) were all detected in response to SARS-CoV2 infection. However, patient plasma showed reduced levels of IFNL1 and/or IFNL2 with increased disease severity.

## Graphical Abstract

**Graphical Abstract:**
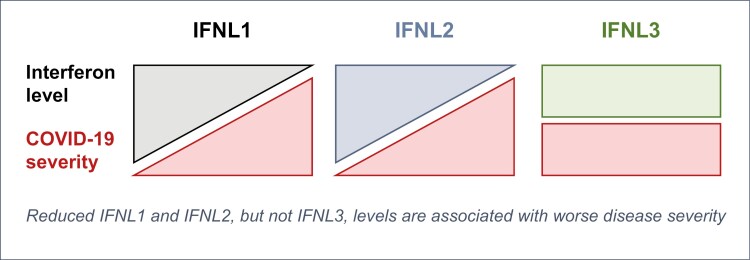

## Introduction

The coronavirus disease 2019 (COVID-19) pandemic caused by severe acute respiratory syndrome coronavirus 2 (SARS-CoV-2) caused global chaos and highlighted the importance of scientific research in fundamental, applied, and clinical immunology. While vaccines were deployed for SARS-CoV-2 within an unprecedented time scale, they only partially prevent viral transmission [1]. Rather, they are highly effective at preventing severe disease caused by a dysregulated immune response to the virus in some individuals. In particular, inadequate early innate immune responses can lead to later over-exuberant responses and severe immunopathology in non-vaccinated individuals [2, 3]. Furthermore, current vaccines provide protection for only a limited time period due to waning immunity. Against a backdrop of emerging viral variants of this new virus, there is an urgent need for improved vaccines with prolonged protective immunity. Understanding the innate and adaptive immune responses that protect, or fail to protect from severe disease, will improve basic understanding of disease pathology and further help tailor new vaccines in terms of a beneficial and prolonged immune response.

The role of type 1 interferon (IFN⍺) in viral infections has long been appreciated. In contrast, much remains to be learned about the role type 3 IFN, or interferon lambda (IFNL) as they are also known. The first three family members, IFNL1 (IL29), IFNL2 (IL28A), and IFNL3 (IL28B) were discovered in 2003 and their genes encode for soluble cytokines that play an important role in mucosal tissues, in particular intestinal epithelia where they confer protection against viral pathogens such as rotavirus [4–6]. Less is known about IFNL4 which was only discovered in 2013 [7]. IFNL4 is characterized by allelic diversity, including a dimorphism that determines either a full length or a truncated non-expressed variant. These genes first came to prominence in Hepatitis C virus (HCV) research where single nucleotide polymorphisms (SNPs) associated with IFNL genes were strongly predictive of spontaneous HCV resolution [8, 9].

However, the data emerging on type 3 family of IFNs in SARS-CoV-2 infection is confusing and much of it is contradictory. There are many reasons for this, including the use of interferon stimulated genes (ISG)s or transcription of IFNL cytokines as readouts of IFNL production (rather than protein production) [10, 11], differential expression of cytokines in different tissues [2, 10, 12], variation in cohorts and timings of samples, and indeed, the conflation of three cytokines (IFNL1, IFNL2, and IFNL3) into generic IFNL in some studies which limits knowledge on potentially important differences in these cytokines and the role they may play in the immune response to SARS-CoV-2 [2, 3]. In general, there is convincing evidence that type 3 IFN are expressed in the airways in response to SARS-CoV-2 infection but data on circulating levels of IFNL in patients with COVID-19 are more variable with conflicting reports in terms of induction and their association with disease progression [2, 3, 13–16].

In this study, we therefore conducted an extensive study of circulating levels of IFNL1, IFNL2, and IFNL3, in addition to SNP typing two loci in the IFNL genomic region, in a large cohort of well characterized, genetically homogenous Irish COVID patients. This cohort was particularly useful as it had good representation from non-hospitalized COVID-19 patients thereby providing important information on characteristics of a beneficial immune response that results in a milder disease outcome. We were able to expand our genetic association results in a second cohort of more ethnically diverse UK patients with more detrimental clinical outcomes. Finally, we were able to test the hypothesis that IFNL cytokines are generally beneficial against SARS-CoV-2 in our cohort by investigating associations between type 3 IFNL protein levels in blood with clinical outcomes.

## Materials and methods

### Study cohorts

PCR-positive SARS-CoV-2 patients were recruited in two cohorts. The first, from St. James’s Hospital, Dublin 8, Ireland comprised 399 patients from whom EDTA plasma was available, and 319 patients (partially overlapping) from whom genomic DNA was available. The cohort was unvaccinated, with the exception of *n* = 3 participants who were recruited after vaccine roll out in Ireland. Patients with Hepatitis C infection had previously been recruited in St. James’s Hospital and plasma stored at −80°C [17] and healthy controls were recruited within Trinity College where blood was taken by a trained phlebotomist.

Clinical severity was scored according to the World Health Organization (WHO) Clinical Progression score (see Supplementary Fig. S2). The Irish cohort included a significant proportion (46%) of individuals with mild disease (WHO score 1–2) that did not require hospital admission. The cohort was predominantly White (84%) and female (60%), and the majority (54%) were hospitalized with mild/moderate disease (WHO score ≥ 3; Table 1 and Supplementary Table S1). Samples from the Irish cohort were collected across a range of sample time-points (d0–d255) after diagnosis and this information was available for 278 participants (Fig. 3e for numbers in each group). Ethics for the Irish study were granted by Tallaght and St. James’s Hospital JREC (Ref. JREC 2020-05 List 19 for COVID-19 STTAR samples and 2013/05/03 List 43 for HCV samples). Ethics for healthy controls was from the STEM Ethics committee in Trinity College Dublin (Ref:TCDFEMSREC_ 02032020_Gard_Slat).

**Table 1:** Overall patient cohort demographics

	Irish	UK
Number of patients (gDNA)	319	242
Number of patients (plasma)	399	56
Non-hospitalized (WHO 1–2)	46%	0
Hospitalized (> WHO 3)	54%	100%
% Female	60%	48%
Ethnicity	White: 87% Asian: 11% Black: 2%	White: 35% Asian: 19% Black: 38% Other/NR: 30%
Median age	56	65
Age range	19–95	21–95
Sample DfSO < 7 days	36% (*n* = 278)	64% (*n* = 56)

The second cohort was recruited from patients at St George’s Hospital, London, UK as part of the ‘Development and Assessment of Rapid Testing for SARS-CoV02 Outbreak’ (DARTS) study (NCT04351646). It comprised 242 patients from whom gDNA was analysed and 56 from whom plasma was analysed. This cohort included more patients with severe disease and was more ethnically diverse (see Table 1). Samples for IFNL analysis were taken early in disease, median 4 days from symptom onset (see Table 1). The DARTS study (NCT04351646) was approved by national HRA and HCRW and Oxford Research Ethics Committee (20/SC/0171).

Both cohorts were well characterized in terms of clinical characteristics and outcome and increased morbidity was associated with older age and higher frailty score, as expected. Most subjects were diagnosed during waves 1 and 2 of SARS-CoV-2 which included both Beta and Delta variants. All study procedures complied with all relevant ethical regulations, following the principles of the Declaration of Helsinki (2008) and the International Conference on Harmonization (ICH) Good Clinical Practice (GCP) guidelines.

### Quantification of IFNL1, IFNL2, and IFNL3

IFNL1, IFNL2, and IFNL3 were measured from 100 μL EDTA plasma using the Duo Set ELISA kit according to manufacturer’s instructions (R&D Systems—DY7246, DY1587, DY5259, respectively). Each biological sample was assayed in duplicate. A standard curve was used for each assay and concentrations derived based on this. Where samples were outside of the linear range of the standard curve, they were diluted and the concentration calculated using the relevant dilution factor. Samples were analysed in batches over sequential days and no particular batch effect was noted. Data was not normalized or standardized between plates.

### IFNL SNP genotyping

Genotyping for the rs8099917 and rs8105790 (IFNL3) was performed by using the rhAmp Genotyping system from Integrated DNA Technologies (IDT) consisting of an rhAmp SNP Assay, rhAmp Genotyping Master Mix and a rhAmp Reporter Mix with Reference. Measurements were done by using a QuantStudio™ 5 Real-Time PCR system for Human Identification Instrument. Quality and outcome of the results was analysed by the QuantStudio Design and Analysis Software.

### Statistical analysis

**Figure 1:**
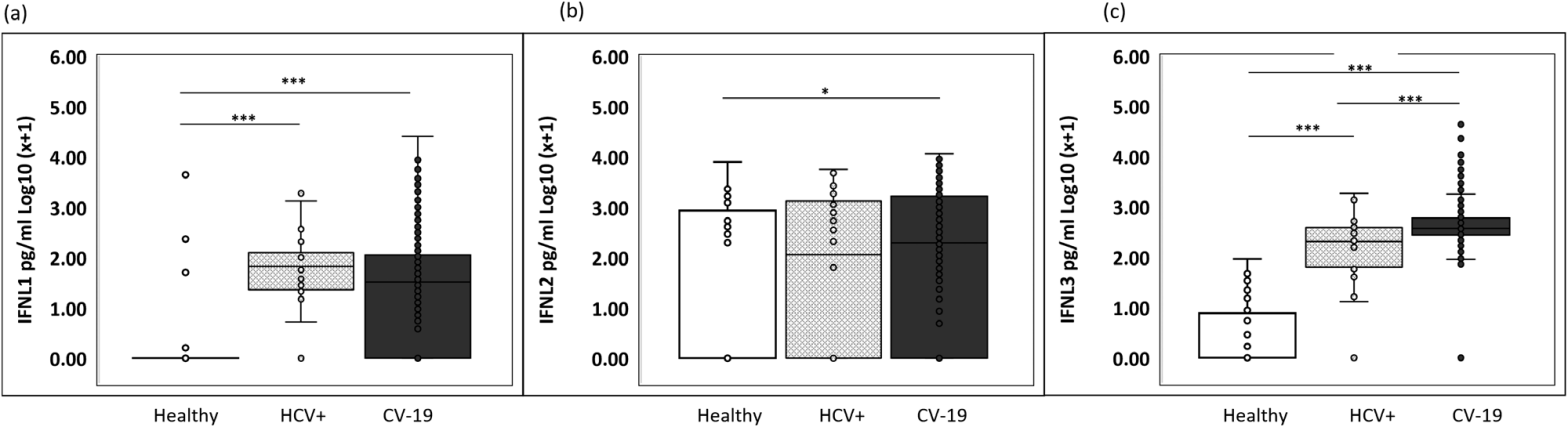
SARS-CoV-2 induces systemic type 3 IFN production. Plasma samples from *n* = 399 Irish patients with COVID-19 (CV-19) were assayed for IFNL1 (a), IFNL2 (b), and IFNL3 (c) by ELISA. Plasma samples from healthy donors (*n* = 35) and HCV + patients (*n* = 26) were also included as indicated. ELISA data was log normalized and box and whisker plots are shown. Samples were analysed by one way ANOVA with Tukey’s test for multiple comparisons. * *P* < 0.05, ***P < 0.001.

**Figure 2:**
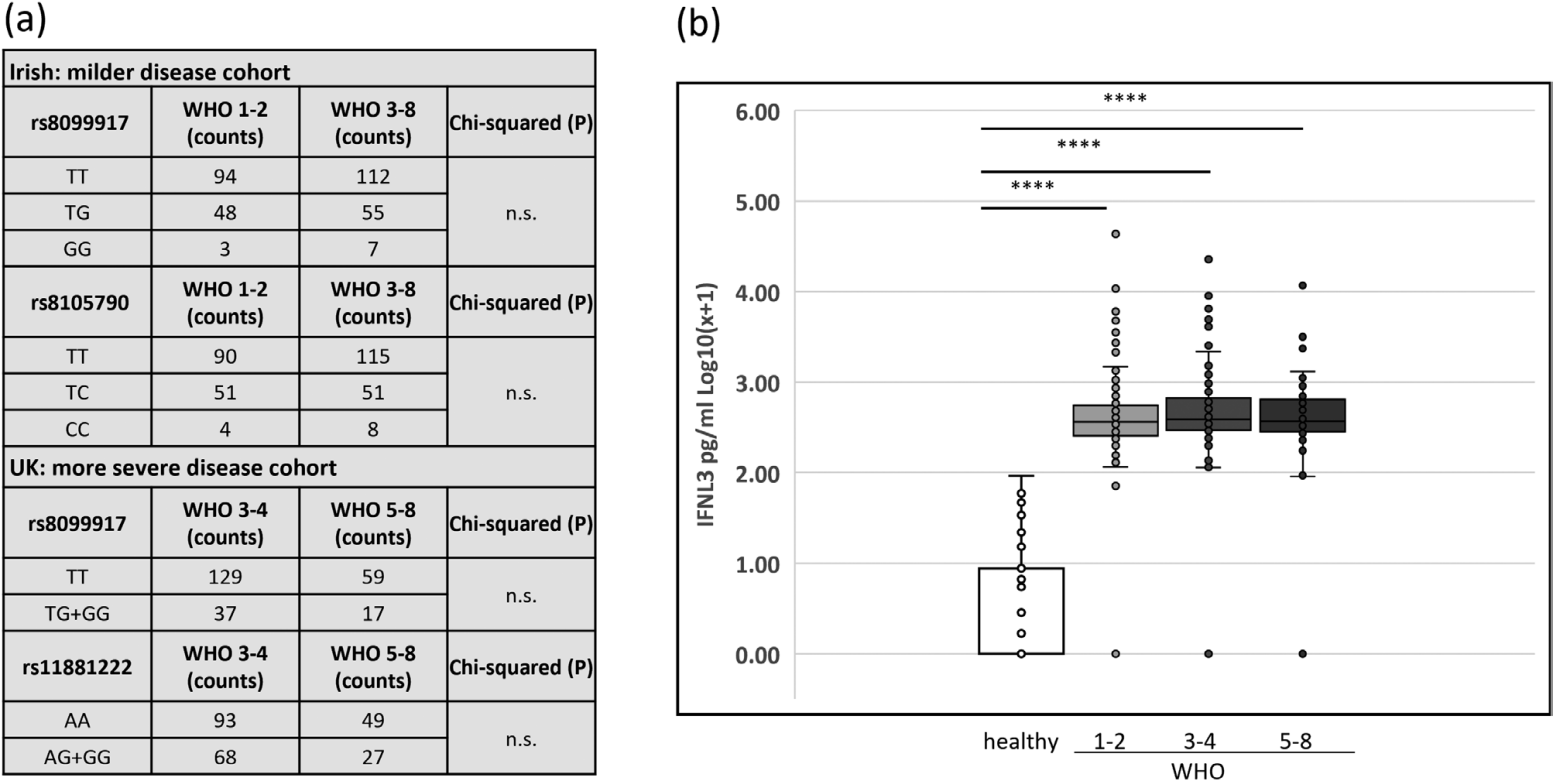
IFNL3 associated SNPs or cytokine levels do not predict severity of infection in COVID-19 patients. gDNA samples from Irish (*n* = 319) and UK (*n* = 242) COVID-19 patients were typed for SNPs including rs8099917, rs8105790, and rs11881222 by qRT-PCR. The cohorts were characterized by differing disease severities and were stratified accordingly for analysis as indicated in the figure. Counts for the various SNP genotypes are shown and Chi-squared analysis performed. n.s. not significant. (b) Cytokine levels were measured by ELISA and plasma levels of IFNL3 for Irish patients with COVID-19 (*n* = 399) were stratified by disease severity into WHO 1–2 (*n* = 183), WHO 3–4 (*n* = 145), and WHO 5–8 (*n* = 72) as indicated. Healthy control IFNL3 (*n* = 35) plasma levels are also included. Data show box and whisker plots for log normalized data which was statistically analysed by a one-way ANOVA with Tukey’s test for multiple comparisons. ****P < 0.0001.

**Figure 3:**
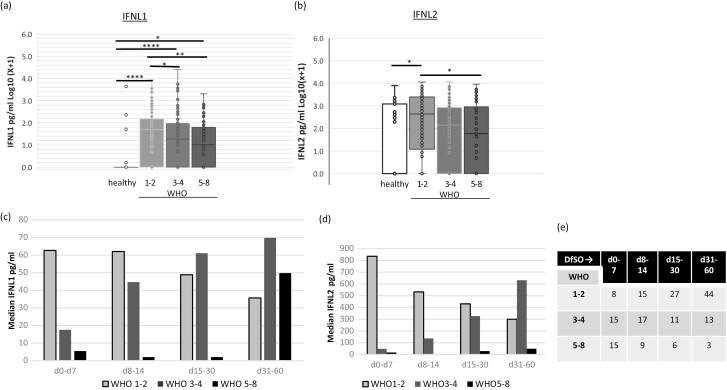
Reduced serum levels of IFNL1 and IFNL2 are significantly associated with worse outcomes. Plasma samples from *n* = 399 Irish COVID-19 patients were assayed for IFNL1 (a) and IFNL2 (b) by ELISA. Plasma samples from *n* = 36 healthy donors were also included as indicated. Patients were stratified based on disease severity as WHO 1–2 (*n* = 183), WHO 3–4 (*n* = 145), or WHO 5–8 (*n* = 72), and box and whisker plots for the data are shown. (a, c) Data was analysed by a one-way ANOVA and Tukey’s test for multiple comparisons. *P < 0.05, **P < 0.01, ****P < 0.0001 (b, d) For a subset of the Irish patients (*n* = 278), information was available to allow further stratification into groups based on sample timing (days from symptom onset, DfSO) which included Days (d) 0–7, d8–14, d15–30 and d30–60 as indicated. Bars show median IFNL levels (pg/ml) and exact numbers in each group are shown in (e).

**Figure 4:**
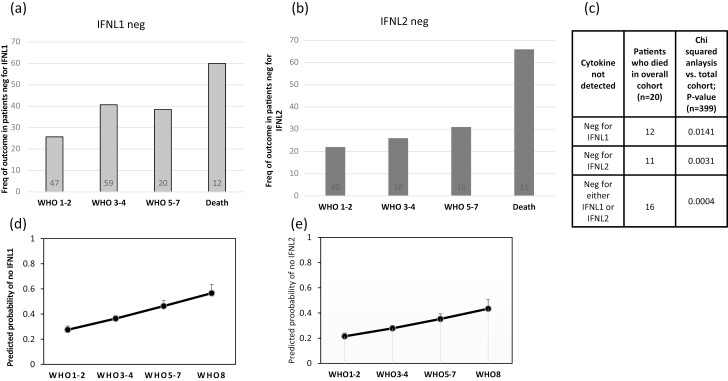
Absence of either IFNL1 or IFNL2 is associated with a worse outcome with COVID-19 patients. Cytokine levels of IFNL1 and IFNL2 were measured from *n* = 399 patients with COVID-19. (a, b) COVID-19 patients who had no detectable IFNL1 (a, *n* = 138) or no detectable IFNL2 (b, *n* = 105) were stratified based on disease severity groups WHO 1–2, WHO 3–4, WHO 5–7, or WHO 8 (death). Bars show the frequency (Freq) of patients that were negative for IFNL in each of a disease severity groups. The number of individuals that contributes to each clinical group is shown inset on each column. (c) For the *n* = 20 patients who died within the overall cohort (*n* = 399), the table shows the counts for patient negative for IFNL1 or IFNL2. Chi-squared analysis was performed relative to the overall cohort and *P*-values shown. Breakdown of other patient outcomes and a representative chi-squared analysis are shown in Supplementary Fig. S4. Panels (d) and (e) show fits from logistic regression models of corresponding data in panels (a) and (b) with the binary response being the presence/absence of IFNL1 and IFNL2 (where absence was the category of interest), and WHO score as a continuous predictor. In each case, AIC analysis indicated that the effect of increasing WHO score across the four categories had a positive and significant effect on the absence of the marker. AIC was 506.25 for IFNL1 model with WHO score vs. 515.74 for a model with intercept only and IFNL2 AIC values were 457.61 vs. 462.72, respectively. Inclusion of age, sex, and smoking status in the models did not alter the conclusions.

A range of statistical tests were used, depending on the data and analysis required. Fig. 1 compared IFNL1, IFNL2, and IFNL3 levels in three separate cohorts: the overall Irish cohort (*n* = 399), healthy donors (*n* = 35), and HCV + donors (*n* = 26). A one-way ANOVA with a Tukey test was used. *P < 0.05, ***P < 0.001. In general, GraphPad PRISM version 9 (GraphPad Software, Inc.) was used for data analysis. In Fig. 2a Chi-squared analysis was performed to test for increased or decreased frequency of particular IFNL3 associated SNPs. In the upper part of the table, 319 Irish COVID patients were tested while analysis of 242 patients from the UK cohort is shown in the lower part of the table. In Fig. 2b, a one-way ANOVA with Tukey test was used to test for differences from the Irish cohort (*n* = 399) patients stratified by WHO 1–2 (*n* = 183), WHO 3–4 (*n* = 145), and WHO 5–8 (*n* = 72). Serum from healthy donors (*n* = 35) was included also as a negative reference control. In Fig. 3a and b, a one-way ANOVA with Tukey test was used for *n* = 399 Irish patients stratified by WHO 1–2 (*n* = 183), WHO 3–4 (*n* = 145), WHO 5–8 (*n* = 72), and healthy controls (n = 35). No statistical test was performed for Fig. 3c and d as numbers became limiting for some groups upon stratification. Fig. 4 used data on patients from the Irish cohort (*n* = 399) that were negative in their serum for either IFNL1 (*n* = 138 patients) or IFNL2 (*n* = 105). The counts for each condition are shown inset on the columns. We fitted logistic regression models to the data in panel (a) and (b) of Fig. 4, with the binary response being the presence/absence of cytokine (absence was the category of interest) and the WHO score as a continuous predictor. AIC analysis was performed (Fig. 4d and e). Chi-squared analysis for IFNL1 neg, IFNL2 neg or negative for either IFNL1 or IFNL2, was performed on those patients (*n* = 20) that died as a result of COVID. The overall cohort of *n* = 399 was used to calculate expected frequencies.

## Results

### SARS-CoV-2 induces systemic type 3 IFN production

To quantify levels of type 3 interferons in our cohort of Irish patients, we measured IFNL1 (IL29), IFNL2 (IL28A), and IFNL3 (IL28B) levels from plasma of *n* = 399 patients with PCR-confirmed SARS-CoV-2. We included plasma from 35 healthy individuals and 26 HCV + patients as reference controls (Fig. 1). There was considerable qualitative and quantitative variation observed both between cytokines, and the different viral infections. While IFNL1 (IL29) was not detected in plasma from most healthy donors, it was significantly increased in plasma of both COVID-19 and HCV + patients (Fig. 1A). One COVID-19 patient (with many risk factors) had extremely high levels (25 410 pg/ml) of IFNL1 (see Table 2 for summary of cytokine measurement data). In the overall cohort, however, only 65% of Irish COVID-19 patients had measurable IFNL1 cytokine, and the median level of IFNL1 was relatively low at 30 pg/ml (Table 2). A higher frequency of HCV + patients had measurable IFLN1 (85%) with a median measurement of 60 pg/ml (Table 2). Overall, IFNL1 was present at lowest amounts and at the lowest frequency in the Irish COVID patients compared to IFNL2, or IFNL3 which had highest levels and frequency overall (see Supplementary Fig. S1 and Table 2).

**Table 2:** Plasma levels of IFNL1, IFNL2, and IFNL3 in cohort of patients with COVID-19

	IFNL1 (IL29)			IFNL2 (IL28A)			IFNL3 (IL28B)		
	Healthy (*n* = 35)	HCV (*n* = 26)	COVID (*n* = 399)	Healthy (*n* = 35)	HCV (*n* = 26)	COVID (*n* = 399)	Healthy (*n* = 35)	HCV (*n* = 26)	COVID (*n* = 399)
Freq (% pos)	14.3%	84.6%	65%	48.6%	53.8%	75.0%	37.0%	95.5%	95.0%
Average of total (pg/ml)	135.5	199.6	276.8	721.8	987.3	1255.9	9.9	314.9	918.4
Median of total (pg/ml)	0	66.1	29.7	0	133.8	193.3	0	203.4	370.7
Median of positives (pg/ml)	56.4	101.5	70.3	606.7	1261.0	661.0	17.6	210.0	383.4
Min/max (pg/ml)	0/230	0/1885	0/25,410	0/2298	0/5576	0/11,530	0/91.5	0/1374	0/43,100

IFNL2 had quite a different profile with roughly similar frequencies of healthy controls (48.6%) and HCV + plasma samples (53.8%) having detectable levels of this cytokine; this increased to 75% of the COVID-19 patients (Fig. 1B and Table 2). COVID-19 positive patients also had higher median levels (193.3 pg/ml), compared to HCV + controls (133.9 pg/ml) and a median of zero was recorded for healthy controls (see Table 2). Viral infection was more consistently associated with IFNL3 with approximately 95% of patients with either SARS-CoV-2 or HCV having detectable amounts of plasma cytokine compared with 37% of healthy donors. The median amount of IFNL3 was highest for patients with COVID-19 (Fig. 1C and Table 2). IFNL3 plasma levels were also found at highest frequency and median levels in the smaller cohort of UK COVID-19 patients (Supplementary Fig. S1). Therefore, all cytokines were detected and were increased in either frequency or amount during viral infection. While only IFNL3 had significantly higher levels of cytokine compared to HCV + patients, both IFNL1 and IFNL2 were significantly higher in COVID-19 patients compared to healthy controls.

### IFNL3/4 associated SNPs do not predict severity of infection in COVID-19 patients

Given that SNPs associated with the IFNL genomic region predict beneficial immune responses in HCV infection, we tested whether SNP rs8105790 (upstream of IFNL3 in an intergenic region), rs8099917 (downstream of IFNL4), or rs11881222 (IFNL3 gene intron) might be predictive of outcome in our COVID cohorts [7]. To first address whether IFNL SNPs predicted mild vs. a more severe disease requiring hospitalization (WHO classification 1–2 ≥  3, see Supplementary Table S1), we typed gDNA from 321 Irish COVID patients for rs8099917 (T/G) and rs8105790 (T/C). Chi-squared analysis showed that neither SNP predicted outcome for patients infected with SARS-CoV-2 (see Fig. 2a). Given that the UK cohort had more patients with severe disease (all 241 patients were admitted to hospital), we further addressed whether rs8099917 (T/G) or rs11881222 (A/G) were associated with the requirement for ventilatory support (WHO ≥ 5; non-invasive ventilation/high flow oxygen or intubation and ventilation). However, neither SNP predicted the need for ventilatory support (Fig. 2a).

### IFNL3 cytokine levels do not predict severity of infection in COVID-19 patients

We next stratified data from the Irish patient cohort to investigate if IFNL3 cytokine levels could predict prognosis within hospitalized COVID-19 patients (*n* = 399). However, levels were similar in patients with good (WHO 1–2), mild (WHO 3–4), and bad (WHO 5–8) clinical outcomes (Fig. 2b), which was consistent with the SNP data. To look at kinetics of production of IFNL3 in the Irish cohort for whom we had relevant information (*n* = 278 patients), we stratified data based on sample time (days) from symptom onset (DfSO). There was early production of IFNL3 in all patients (Days 0–7 in Supplementary Figs. S2 and S3c) but overall, there were no associations between IFNL3 production and disease severity in any of the differing disease outcome groups. It was interesting to observe that IFNL3 remained at elevated levels for up to 3–6 months, even in those patients with very mild COVID-19. Thus, although circulating IFNL3 is produced and sustained in response to SARS-CoV-2 infection, it was not predictive of disease progression.

### Reduced or absent circulating IFNL1 and IFNL2 levels are associated with more severe COVID outcome

Finally, we investigated if IFNL1 or IFNL2 levels were associated with disease severity in COVID-19 patients using good (WHO 1–2), mild (WHO 3–4), and bad (WHO 5–8) clinical outcomes to stratify the large cohort of Irish patients (*n* = 399). IFNL1 was found in progressively lower amounts with increasing disease severity (WHO1–2, 3–4, and 5–8; Fig. 3a). Although levels were generally quite low, IFNL1 was produced early with highest median levels at the earlier sampling points (up to two weeks, Fig. 3c and e and Supplementary Fig. S3a) in patients with mild disease; while the lower patient numbers in the stratified data preclude statistical analysis, the trend suggests that higher levels at earlier times may be protective. There was a potential delay in production in the WHO 3–4 group in which the median increased over time.

Lower IFNL2 was also associated with a more severe clinical outcome in COVID-19 patients (Fig. 3b) with significantly reduced levels in patients with severe disease (WHO 5–8) compared to a milder outcome (WHO 1–2). Similar to trends seen for IFNL1, stratification of patients for temporal analysis found that IFNL2 cytokine was present very early with the highest median value recorded at the first sampling time frame of 0–7 days (Fig. 3d,e, Supplementary Fig. S3b). Thereafter, the median gradually decreased in the mild patient group for samples up to 2 months. This pattern contrasted with the WHO 3–4 (good outcome) group in which the median levels started low and gradually increased up to about 2 months.

Both IFNL1 and IFNL2 levels were consistently low in patients with most severe COVID-19 (WHO 5–8). It was striking to note that a complete absence of either IFNL1 or IFNL2 occurred more frequently with more severe COVID-19. Stratification of 138 COVID-19 patients without any detectable IFNL1 into groups based on disease severity demonstrated a clear association between absence of IFNL1 and worse outcome (Fig. 4a). Indeed, of the 20 patients in the cohort who died of COVID-19, 12 had no detectable IFNL1 (60%) compared to the overall cohort (35%). Performing a similar analysis on the 75 COVID-19 patients with no plasma IFNL2 detectable (Fig. 4b), there was a clear association between worse outcome and absence of IFNL2 with 11 of the 20 patients who died (55%) having no detectable IFNL1 compared to 25% of the overall cohort. Of note, the vast majority of patients who died (16 of 20) lacked either IFNL1 or IFNL2 (Fig. 4c).

Logistic regression modelling (using AIC values for the model with WHO score specified as a continuous variate vs. as a factor) confirmed that the probability that IFNL1 was absent increased significantly across the WHO score (Fig. 4d and using raw data from Fig. 4a) with similar findings for IFNL2 (Fig. 4e, with raw data from Fig. 4b).

Thus, understanding the biology of IFNL1 and IFNL2 and their role in protection against severe SARS-CoV-2 infection is an important research focus.

## Discussion

Defining what constitutes a beneficial immune response to SARS-CoV-2 is important for understanding mechanistic, prognostic, therapeutic, and vaccine development aspects of the disease. Although interferons are recognized as key anti-viral regulators, the data emerging are complex and often contradictory [15]. In this study, we have focussed on type 3 IFNL in a large cohort (~500 patients) of Irish patients which includes important representation from patients with mild disease (WHO 1–2). Our data highlights the importance of considering type 3 IFNs as a family of cytokines within which individual cytokines can play independent roles. Indeed, we found qualitative and quantitative differences in terms of frequency of induction, amount of cytokine produced and associations with severe COVID-19 outcome among IFNL1, IFNL2, and IFNL3. All three cytokines were induced in plasma in response to SARS-CoV-2 infection as was expected of innate anti-viral cytokines. IFNL3 was induced in 95% of COVID-19 patients and at higher median levels compared to the IFNL1 or IFNL2. Its production profile was similar to that seen in a control HCV cohort where it was also detected in 95% of patients, albeit at lower median levels (Table 2). It was interesting to observe that IFNL3 levels were maintained, even in patients who had very mild disease, but were not associated with outcome of COVID-19. It is not clear what function IFNL3 serves in the context of SARS-CoV-2 infection but the sustained levels of IFNL3 across all disease severity groups suggest that it is worth investigating a potential role in long-COVID which can impact patients, regardless of severity of initial infection.

We tested for two SNPs in the IFNL genomic region that were previously identified to strongly predict both spontaneous resolution of HCV and patient response to IFNα therapy. As mentioned, rs8105790 did not predict prognosis of SARS-CoV-2 infection in our cohort. The older literature is confusing regarding which genes particular SNPs mark and how they might relate to any functional impact. In particular SNPs that are now known to better associate with IFNL4, including rs8099917 (located in an intergenic region closest to IFNL4), paradoxically predict a non-expressed beneficial IFNL variant in HCV infection [18, 19]. While rs8099917 TT genotype has been reported to be beneficial in a cohort of Iranian COVID-19 patients [20], we found no association in either Irish or a mixed ethnicity UK cohort. However, rs8099917 is in relatively weak linkage disequilibrium with IFNL4 functional SNPs in European populations (such as Irish [7],) and the lack of association should be confirmed with other informative SNP e.g. rs368234815 before ruling out a role for IFNL4 in COVID-19 patients. The natural population variation of informative SNP frequencies means that genetic and/or biological associations may have limited use for COVID-19 in a global context.

Of more clinical interest was the observation that IFNL1 and IFNL2 were produced in 65% and 75%, respectively of patients with COVID and production was associated with better outcomes in this cohort. This is important in the context of trying to understand whether IFNL are beneficial or perhaps detrimental in COVID-19, as previously raised by some studies [15]. In our study, patients with mild COVID had a rapid production of IFNL1 and IFNL2, which although decreasing over time, was still detectable 2 months post-infection. This contrasted starkly with patients who had a more severe COVID-19 disease trajectory. Similarly, when patients who lacked these cytokines were stratified by outcome, we observed a clear negative correlation between cytokine levels and disease severity, suggesting that the absence of IFNL1 or IFNL2 may contribute to disease progression. There may be some redundancy in the roles these cytokines play based on the observation that lacking either IFNL1 or IFNL2 was detrimental, and these phenotypes were overrepresented in the patients who died in our study. As we only have one sample per patient, the absence of a cytokine at a particular time does not mean that the cytokine was never there. However, the patients who died in our cohort had samples across the range of times and not one particular time point. Our data are consistent with a model in which, for this genetically homogenous population, both IFNL1 and IFNL2 production are beneficial in SARS-Co2 infection and furthermore, a failure to make IFNL1 and/or IFNL2 at earlier time points hampers the ability of the immune response to clear the virus. The potential use of IFNL as a biomarker(s) of severe COVID-19 illness deserves further investigation.

There are some limitations to our study including that while we have a large number of samples at a range of well documented time points, they are not serial samples from the same patients which would give a more accurate picture of induction and fluctuation profiles. Stratification to look at temporal patterns of cytokine levels also reduced the numbers available in each group and while most were fine, some had lower numbers and will need validation in other studies. Finally, we had access to peripheral blood only for this study and some reports have shown differences in IFNL between airways (the primary site of infection) and blood, which we were unable to investigate.

Overall, the patterns observed for IFNL2 and IFNL1 (and IFNL3 to a lesser extent) were similar and consistent with the currently accepted pattern of type I IFN during COVID-19 [11, 21, 22]. In particular, early production is beneficial but severe disease is characterized by an absence of cytokine. However, association is not causation and in our study, as in others, it is not clear whether the reduced cytokine levels in severe disease patients are caused by viral encoded mechanisms or indeed, whether individuals that only make low/no IFNL2 or IFNL1 for reasons, e.g. genetic polymorphism are more susceptible to severe SARS-CoV-2. Further work is required to dissect possible mechanisms and it is likely that both host and virus will contribute to the clinical phenotype observed.

## Supplementary data

Supplementary data is available at *Clinical and Experimental Immunology* online.

uxae047_suppl_Supplementary_Data

## Data Availability

The authors confirm that the data supporting the findings of this study are available within the article [and/or] its Supplementary materials.

## Abbreviations:

HCV: Hepatitis C virus
IFNL: interferon lambda
ISG: interferon stimulated genes
SARS-CoV-2: severe acute respiratory syndrome coronavirus 2;
SNPs: single nucleotide polymorphisms.

## References

[1] Franco-Paredes C. Transmissibility of SARS-CoV-2 among fully vaccinated individuals. Lancet Infect Dis 2022, 22, 16. doi:10.1016/S1473-3099(21)00768-4 PMC8694744 34953540

[2] Blanco-Melo D , Nilsson-Payant BE, Liu WC, Uhl S, Hoagland D, Moller R, et al Imbalanced host response to SARS-CoV-2 drives development of COVID-19. Cell 2020, 181, 1036–45.e9. doi:10.1016/j.cell.2020.04.026 32416070 PMC7227586

[3] Lucas C , Wong P, Klein J, Castro TBR, Silva J, Sundaram M, et al; Yale IMPACT Team. Longitudinal analyses reveal immunological misfiring in severe COVID-19. Nature 2020, 584, 463–9. doi:10.1038/s41586-020-2588-y 32717743 PMC7477538

[4] Stanifer ML , Guo C, Doldan P, Boulant S. Importance of type I and III interferons at respiratory and intestinal barrier surfaces. Front Immunol 2020, 11, 608645. doi:10.3389/fimmu.2020.608645 33362795 PMC7759678

[5] Egli A , Santer DM, O’Shea D, Tyrrell DL, Houghton M. The impact of the interferon-lambda family on the innate and adaptive immune response to viral infections. Emerg Microbes Infect 2014, 3, e51. doi:10.1038/emi.2014.51 26038748 PMC4126180

[6] Zhou JH , Wang YN, Chang QY, Ma P, Hu Y, Cao X. Type III interferons in viral infection and antiviral immunity. Cell Physiol Biochem 2018, 51, 173–85. doi:10.1159/000495172 30439714

[7] Fang MZ , Jackson SS, O’Brien TR. IFNL4: notable variants and associated phenotypes. Gene 2020, 730, 144289. doi:10.1016/j.gene.2019.144289 31846709 PMC8600600

[8] Suppiah V , Moldovan M, Ahlenstiel G, Berg T, Weltman M, Abate ML, et al IL28B is associated with response to chronic hepatitis C interferon-alpha and ribavirin therapy. Nat Genet 2009, 41, 1100–4. doi:10.1038/ng.447 19749758

[9] Tanaka Y , Nishida N, Sugiyama M, Kurosaki M, Matsuura K, Sakamoto N, et al Genome-wide association of IL28B with response to pegylated interferon-alpha and ribavirin therapy for chronic hepatitis C. Nat Genet 2009, 41, 1105–9. doi:10.1038/ng.449 19749757

[10] Sposito B , Broggi A, Pandolfi L, Crotta S, Ferrarese R, Sisti S, et al Severity of SARS-CoV-2 infection as a function of the interferon landscape across the respiratory tract of COVID-19 patients. bioRxiv 2021. 10.1016/j.cell.2021.08.016 PMC8373821 34492226

[11] Combes AJ , Courau T, Kuhn NF, Hu KH, Ray A, Chen WS, et al; UCSF COMET Consortium. Global absence and targeting of protective immune states in severe COVID-19. Nature 2021, 591, 124–30. doi:10.1038/s41586-021-03234-7 33494096 PMC8567458

[12] Zhou Z , Ren L, Zhang L, Zhong J, Xiao Y, Jia Z, et al Heightened innate immune responses in the respiratory tract of COVID-19 patients. Cell Host Microbe 2020, 27, 883–90.e2. doi:10.1016/j.chom.2020.04.017 32407669 PMC7196896

[13] Galani IE , Rovina N, Lampropoulou V, Triantafyllia V, Manioudaki M, Pavlos E, et al Untuned antiviral immunity in COVID-19 revealed by temporal type I/III interferon patterns and flu comparison. Nat Immunol 2021, 22, 32–40. doi:10.1038/s41590-020-00840-x 33277638

[14] Venet M , Ribeiro MS, Decembre E, Bellomo A, Joshi G, Nuovo C, et al Severe COVID-19 patients have impaired plasmacytoid dendritic cell-mediated control of SARS-CoV-2. Nat Commun 2023, 14, 694. doi:10.1038/s41467-023-36140-9 36755036 PMC9907212

[15] Zanoni I. Interfering with SARS-CoV-2: are interferons friends or foes in COVID-19? Curr Opin Virol 2021, 50, 119–27. doi:10.1016/j.coviro.2021.08.004 34454352 PMC8367741

[16] Fukuda Y , Homma T, Inoue H, Goto Y, Sato Y, Ikeda H, et al Serum IL-28A/IFN-lambda2 is linked to disease severity of COVID-19. Sci Rep 2022, 12, 5458. doi:10.1038/s41598-022-09544-8 35361913 PMC8969403

[17] Keane C , O’Shea D, Reiberger T, Peck-Radosavljevic M, Farrell G, Bergin C, et al Variation in both IL28B and KIR2DS3 genes influence pegylated interferon and ribavirin hepatitis C treatment outcome in HIV-1 co-infection. PLoS One 2013, 8, e66831. doi:10.1371/journal.pone.0066831 23826153 PMC3691248

[18] Hamming OJ , Terczynska-Dyla E, Vieyres G, Dijkman R, Jorgensen SE, Akhtar H, et al Interferon lambda 4 signals via the IFNlambda receptor to regulate antiviral activity against HCV and coronaviruses. EMBO J 2013, 32, 3055–65. doi:10.1038/emboj.2013.232 24169568 PMC3844954

[19] Pedergnana V , Irving WL, Barnes E, McLauchlan J, Spencer CCA. Impact of IFNL4 genetic variants on sustained virologic response and viremia in hepatitis C virus genotype 3 patients. J Interferon Cytokine Res 2019, 39, 642–9. doi:10.1089/jir.2019.0013 31260374 PMC6767867

[20] Rahimi P , Tarharoudi R, Rahimpour A, Mosayebi Amroabadi J, Ahmadi I, Anvari E, et al The association between interferon lambda 3 and 4 gene single-nucleotide polymorphisms and the recovery of COVID-19 patients. Virol J 2021, 18, 221. doi:10.1186/s12985-021-01692-z 34775984 PMC8590865

[21] Hadjadj J , Yatim N, Barnabei L, Corneau A, Boussier J, Smith N, et al Impaired type I interferon activity and inflammatory responses in severe COVID-19 patients. Science (New York, NY) 2020, 369, 718–24. doi:10.1126/science.abc6027 PMC7402632 32661059

[22] Arunachalam PS , Wimmers F, Mok CKP, Perera R, Scott M, Hagan T, et al Systems biological assessment of immunity to mild versus severe COVID-19 infection in humans. Science (New York, NY) 2020, 369, 1210–20. 10.1126/science.abc6261 PMC7665312 32788292

