# Supplementary material for: Reduced IFNL1 and/or IFNL2, but not IFNL3 is associated with worse outcome in patients with COVID-19
Source: Clin Exp Immunol. 2024 Jul 2;218(3):300–7. doi: 10.1093/cei/uxae047 (PMC11557148; doi:10.1093/cei/uxae047)
Supplement: uxae047_suppl_Supplementary_Data [file uxae047_suppl_supplementary_data.zip › IFNL Covid Supplementary Figs revised May 2024.pdf]

Figure S1: Overall IFNL levels in the two Covid-19 cohorts

(a) Irish cohort (n=399)

(b) UK cohort (n=56)

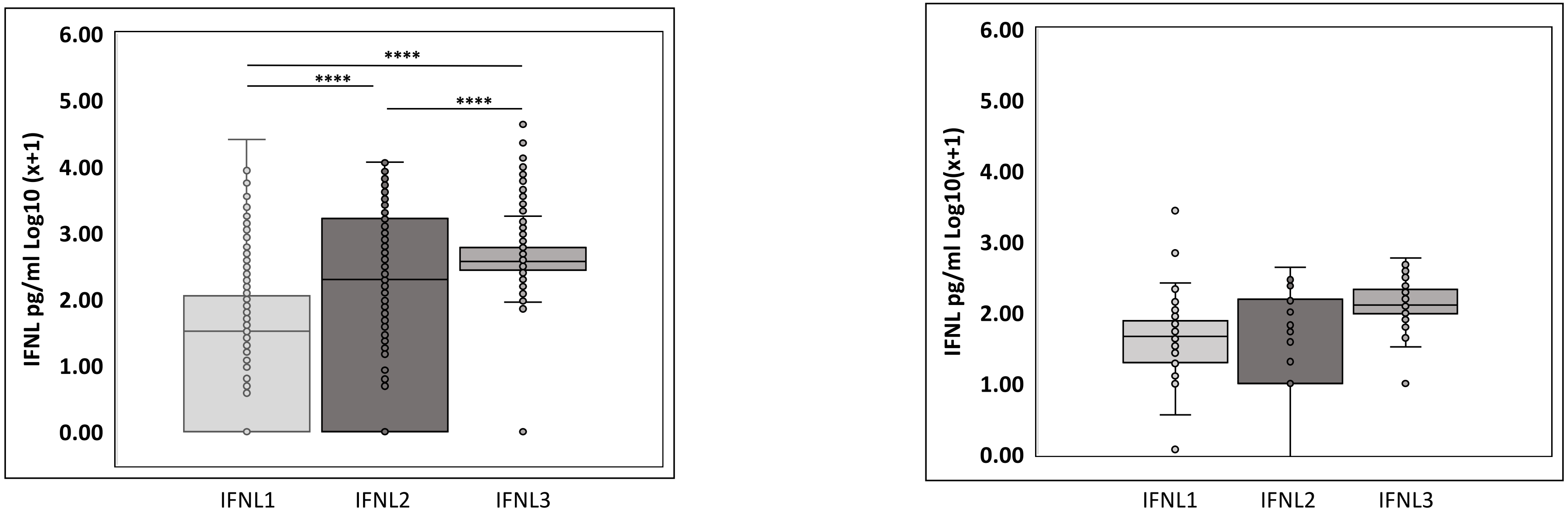

Figure S1. Circulating IFNL1, IFNL2 and IFNL3 cytokines from plasma of Irish (n=399) and UK (n=56) patients with COVID-19. Cytokines were measured by ELISA and pg/ml values log normalized; standard box and whisker plots are shown. Samples were analysed by a paired one way ANOVA with Tukey’s test (a) and one way ANOVA with paired data with mixed effects analysis (b) ****P<0.0001.

Figure S2: Median IFNL3 levels by DfSO and WHO groupings

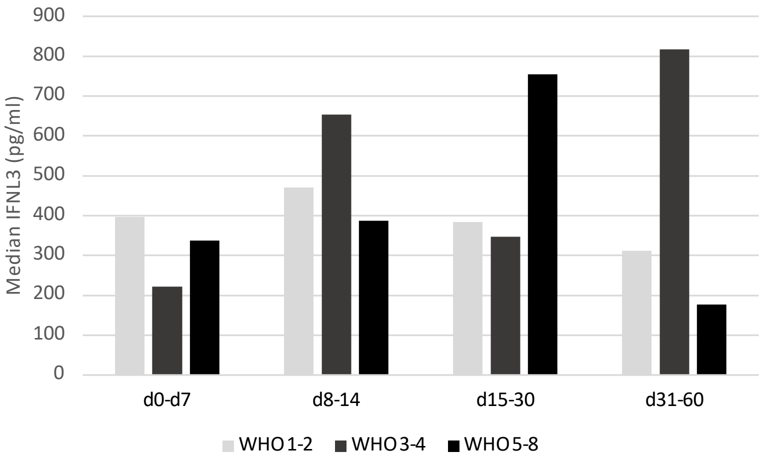

n=183 → DfSO ↓	WHO1-2 (n)	WHO 3-4 (n)	WHO5-8 (n)
d0-d7	8	15	15
d8-d14	15	17	9
d15-d30	27	11	6
d31-d60	44	13	3

Figure S2. IFNL3 was measured from plasma from Irish patients with COVID-19. Patients were stratified for disease severity based on WHO clinical scores: WHO 1-2 (good), WHO 3-4 (mild) and WHO 5-8 (bad) outcomes as indicated by the colour key. Information regarding days from symptom onset (DfSO) was available for n=183 patients, and data was further stratified based on sampling at d0-7, d8-14, d15-30 and d31-60 as indicated on the x-axis. Bars shows the median levels of IFNL while the numbers of samples available for each of these columns is indicated in the Table to the right of the figure.

Figure S3

(a)

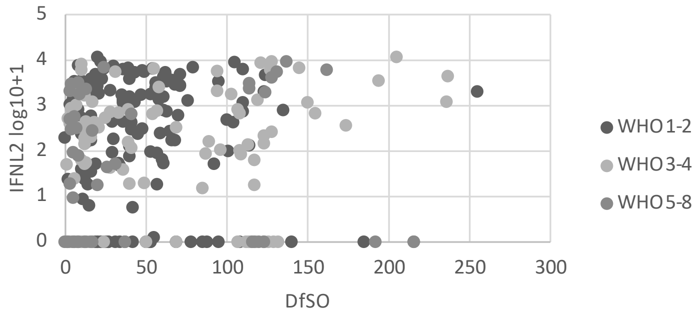

(b)

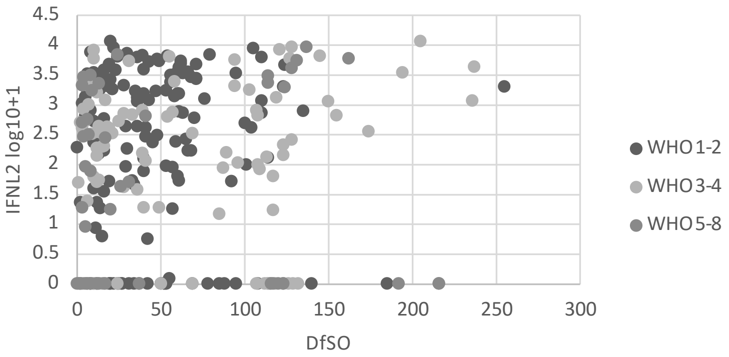

(c)

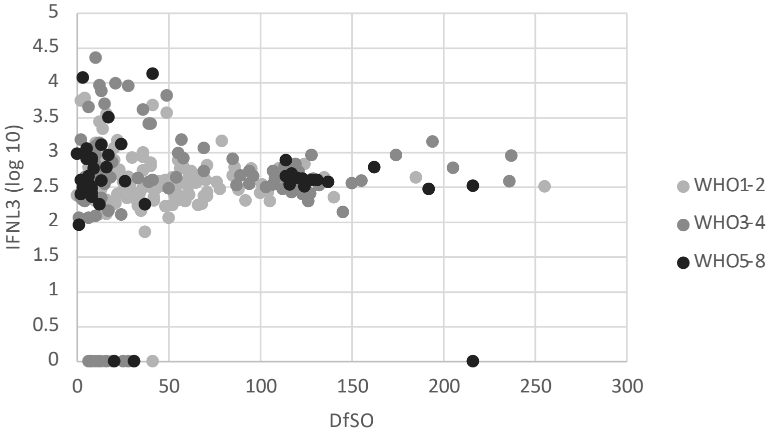

Scatter plots of IFNL1 (a), IFNL2 (b) and IFNL3 (c) by WHO score and by DfSO.

Figure S4: Chi squared analysis of IFNL1 and IFNL2 negative patients within patients who died

Cytokine missing	WHO 1-2 (n=183)	WHO 3-4 (n=145)	WHO 5-7 (n=52)	WHO 8 (n=20)
No IFNL1	47	59	20	12
No IFNL2	40	38	16	11
Missing either IFNL1 or IFNL2	70	48	28	16
Missing both INFL1 and IFNL2	17	19	8	7

	Observed	Expected	Chi-squared P-value
Missing either IFNL1 or IFNL2	16	8.2*	0.0004
Having both IFNL1 and IFNL2	4	11.8	
Total patients who died	20	20	

* Based on the overall cohort of n=399 patients

Table S1: WHO categories for clinical outcomes and numbers of samples per group

(a)

Ordinal Scale for Clinical Improvement

Patient State	Descriptor	Score
Uninfected	No clinical or virological evidence of infection	0
Ambulatory	No limitation of activities	1
	Limitation of activities	2
Hospitalized Mild disease	Hospitalized, no oxygen therapy	3
	Oxygen by mask or nasal prongs	4
Hospitalized Severe Disease	Non-invasive ventilation or high-flow oxygen	5
	Intubation and mechanical ventilation	6
	Ventilation + additional organ support – pressors, RRT, ECMO	7
Dead	Death	8

(b)

Covid patient sample numbers per WHO groupings			
	**Irish cohort**		**UK cohort**
**Sample type**	**Plasma**	**gDNA**	**gDNA**
**Total number**	399	319*	241
**Good outcome (WHO 1-2)**	183	145	0
**Mild outcome (WHO 3-4)**	145	174 (Hospitalised)	166
**Bad outcome (WHO 5-8)**	72		76

*Slightly overlapping
