# Supplementary material for: Reduced IFNL1 and/or IFNL2, but not IFNL3 is associated with worse outcome in patients with COVID-19
Source: Clin Exp Immunol. 2024 Jul 2;218(3):300–7. doi: 10.1093/cei/uxae047 (PMC11557148; doi:10.1093/cei/uxae047)
Supplement: uxae047_suppl_Supplementary_Data [file uxae047_suppl_supplementary_data.zip › IFNL Covid Supplementary Figs revised May 2024.pptx]

## Slide 1
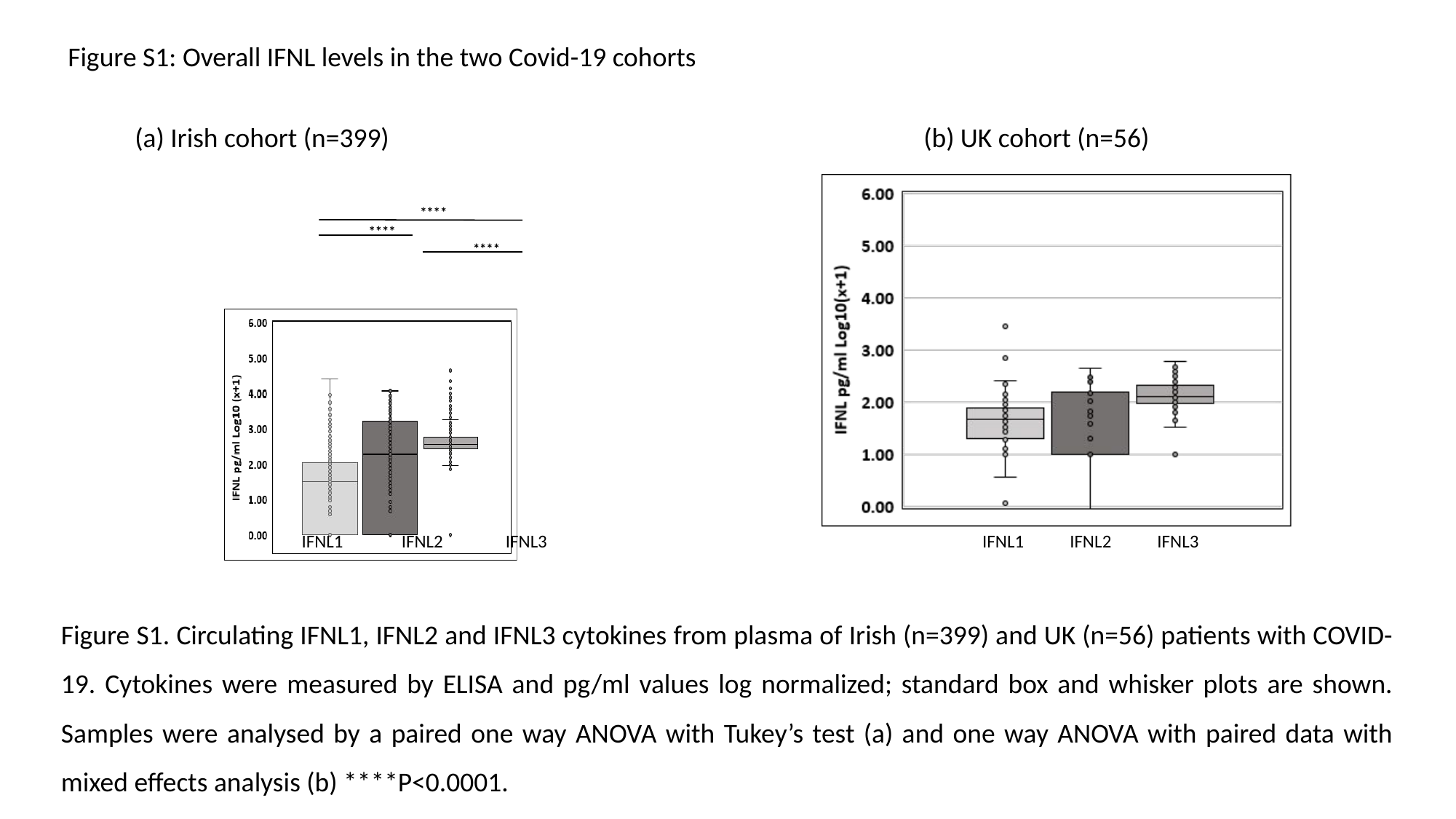

Figure S1: Overall IFNL levels in the two Covid-19 cohorts
(a) Irish cohort (n=399)					 (b) UK cohort (n=56)
****
****
****
IFNL1 IFNL2 IFNL3
IFNL1 IFNL2 IFNL3
Figure S1. Circulating IFNL1, IFNL2 and IFNL3 cytokines from plasma of Irish (n=399) and UK (n=56) patients with COVID-19. Cytokines were measured by ELISA and pg/ml values log normalized; standard box and whisker plots are shown. Samples were analysed by a paired one way ANOVA with Tukey’s test (a) and one way ANOVA with paired data with mixed effects analysis (b) ****P<0.0001.

## Slide 2
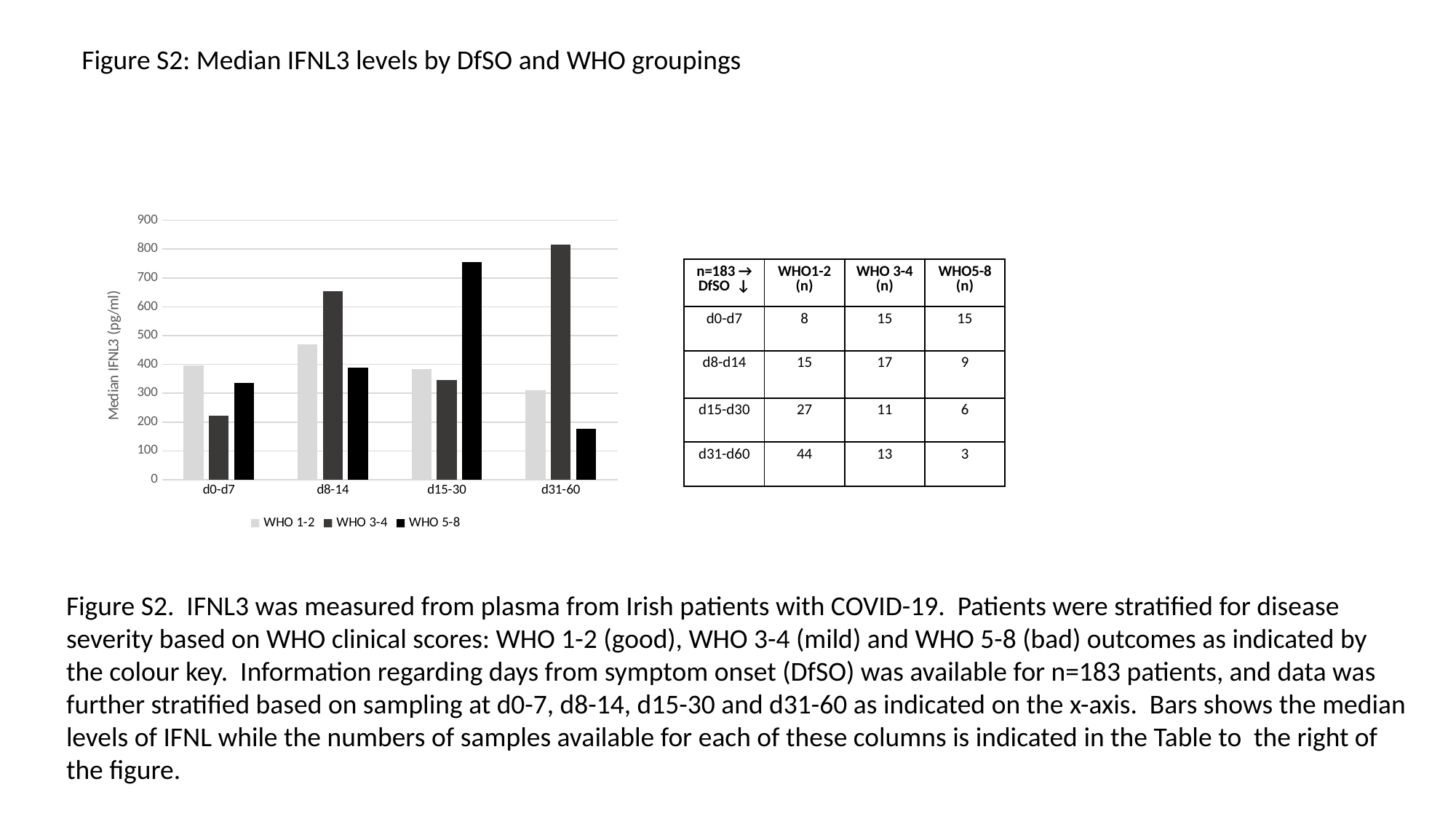

Figure S2: Median IFNL3 levels by DfSO and WHO groupings
### Chart
| Category | WHO 1-2 | WHO 3-4 | WHO 5-8 |
|---|---|---|---|
| d0-d7 | 397.35 | 221.7 | 336.8 |
| d8-14 | 470.8 | 653.1 | 387.8 |
| d15-30 | 383.4 | 346.7 | 754.8499999999999 |
| d31-60 | 311.25 | 816.7 | 177.4 || n=183 → DfSO ↓ | WHO1-2 (n) | WHO 3-4 (n) | WHO5-8 (n) |
| --- | --- | --- | --- |
| d0-d7 | 8 | 15 | 15 |
| d8-d14 | 15 | 17 | 9 |
| d15-d30 | 27 | 11 | 6 |
| d31-d60 | 44 | 13 | 3 |
Figure S2. IFNL3 was measured from plasma from Irish patients with COVID-19. Patients were stratified for disease severity based on WHO clinical scores: WHO 1-2 (good), WHO 3-4 (mild) and WHO 5-8 (bad) outcomes as indicated by the colour key. Information regarding days from symptom onset (DfSO) was available for n=183 patients, and data was further stratified based on sampling at d0-7, d8-14, d15-30 and d31-60 as indicated on the x-axis. Bars shows the median levels of IFNL while the numbers of samples available for each of these columns is indicated in the Table to the right of the figure.

## Slide 3
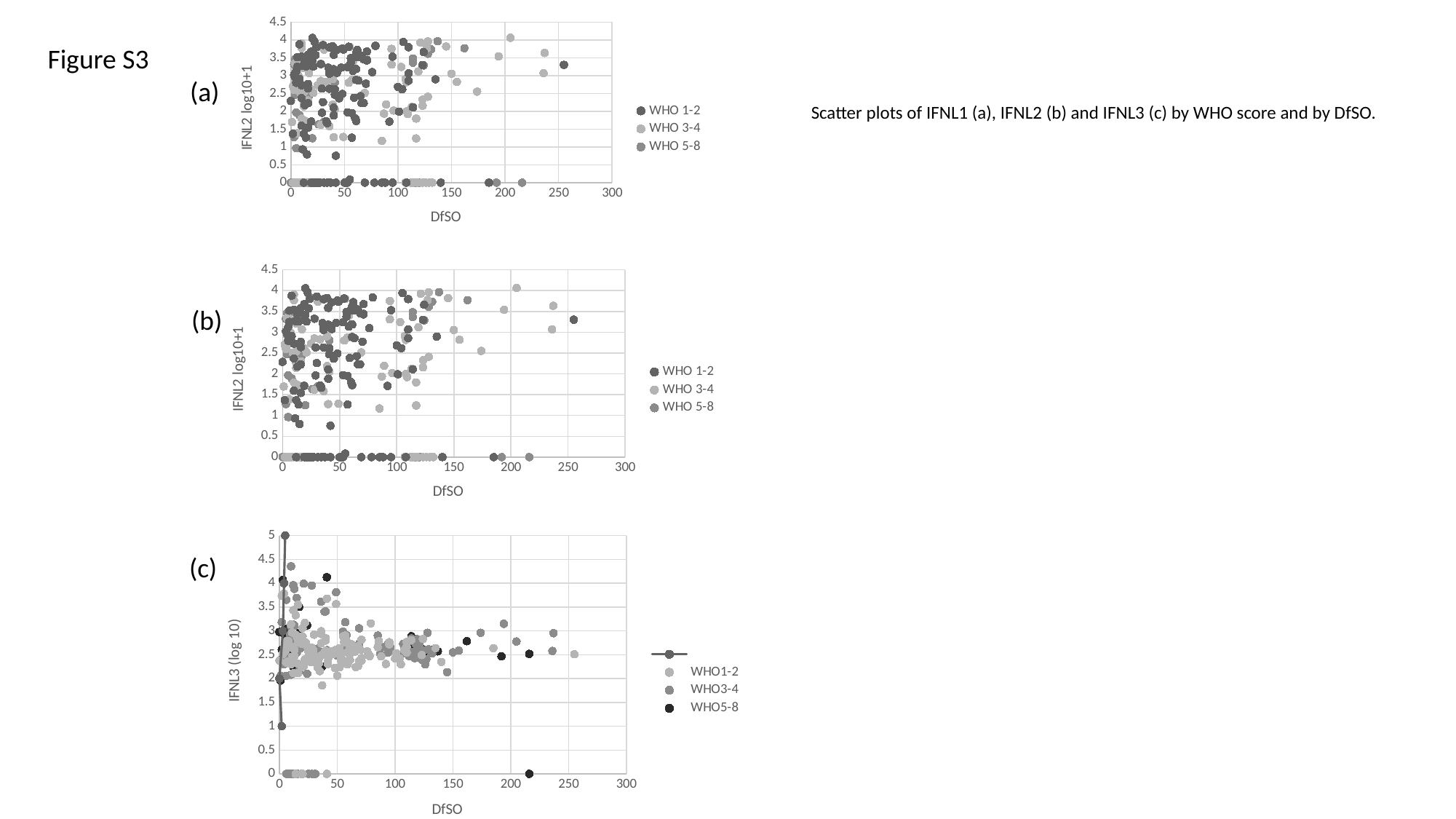

### Chart
| Category | | | |
|---|---|---|---|Figure S3
(a)
Scatter plots of IFNL1 (a), IFNL2 (b) and IFNL3 (c) by WHO score and by DfSO.
### Chart
| Category | | | |
|---|---|---|---|(b)
### Chart
| Category | | | | |
|---|---|---|---|---|(c)

## Slide 4
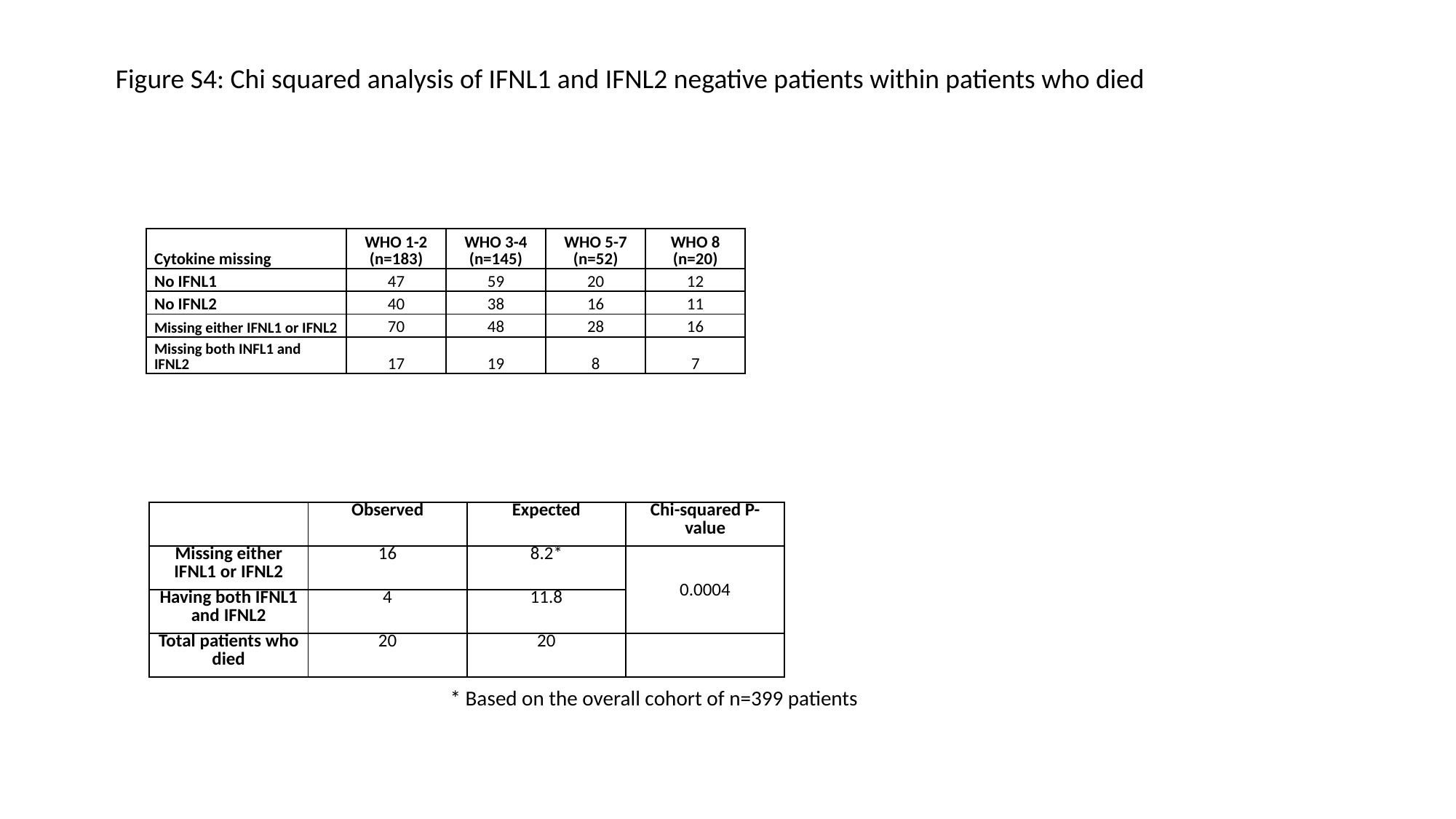

Figure S4: Chi squared analysis of IFNL1 and IFNL2 negative patients within patients who died
| Cytokine missing | WHO 1-2 (n=183) | WHO 3-4 (n=145) | WHO 5-7 (n=52) | WHO 8 (n=20) |
| --- | --- | --- | --- | --- |
| No IFNL1 | 47 | 59 | 20 | 12 |
| No IFNL2 | 40 | 38 | 16 | 11 |
| Missing either IFNL1 or IFNL2 | 70 | 48 | 28 | 16 |
| Missing both INFL1 and IFNL2 | 17 | 19 | 8 | 7 |
| | Observed | Expected | Chi-squared P-value |
| --- | --- | --- | --- |
| Missing either IFNL1 or IFNL2 | 16 | 8.2\* | 0.0004 |
| Having both IFNL1 and IFNL2 | 4 | 11.8 | |
| Total patients who died | 20 | 20 | |
* Based on the overall cohort of n=399 patients

## Slide 5
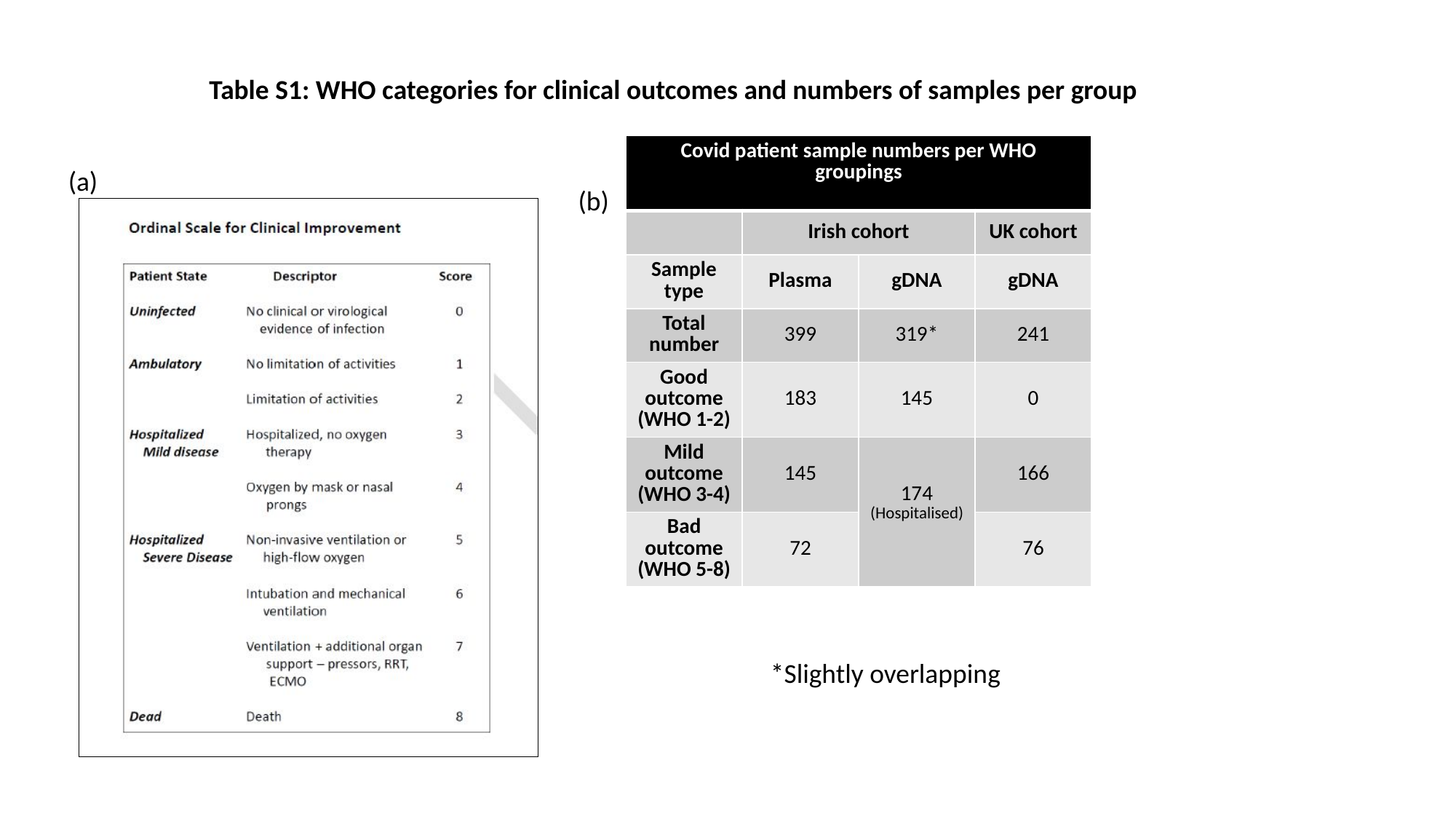

Table S1: WHO categories for clinical outcomes and numbers of samples per group
| Covid patient sample numbers per WHO groupings | | | |
| --- | --- | --- | --- |
| | Irish cohort | | UK cohort |
| Sample type | Plasma | gDNA | gDNA |
| Total number | 399 | 319\* | 241 |
| Good outcome (WHO 1-2) | 183 | 145 | 0 |
| Mild outcome (WHO 3-4) | 145 | 174 (Hospitalised) | 166 |
| Bad outcome (WHO 5-8) | 72 | | 76 |
(a)
(b)
*Slightly overlapping
